# Interfacial regulation of low-intensity acoustic cavitation by MOF-based nanoparticles

**DOI:** 10.1016/j.ultsonch.2026.108064

**Published:** 2026-09-27

**Authors:** Xiaoman Zhang, Jinyu Lin, Qi Zhou, Wenwu Cao

**Affiliations:** Center of Acoustic Functional Materials and Applications, School of Advanced Manufacturing Engineering, Nanjing University, Suzhou 215163, China

**Keywords:** Acoustic cavitation, Low-intensity ultrasound, Cavitation threshold, ZIF-8 nanoparticles, Interfacial engineering

## Abstract

Ultrasound-induced cavitation plays an important role in ultrasound-mediated treatments, drug delivery, and sonodynamic therapy, yet the mechanism of regulatory functions of metal–organic framework (MOF) nanoparticles toward cavitation intensity is still not fully understood. In this study, zeolitic imidazolate framework-8 (ZIF-8) was used as a model system to investigate the effects of particle size, external morphology, and mesoporous silica shell engineering on cavitation behavior under low-intensity ultrasound. The results showed that bare ZIF-8 nanoparticles exhibited limited cavitation enhancement, but displayed a clear non-monotonic size dependence, with the intermediate-sized sample showing the lowest apparent cavitation threshold. Comparison between rhombic dodecahedral and cubic ZIF-8 further indicated that the external geometric boundary of the particles affects their nonlinear acoustic response. Compared with size and morphology regulation alone, mesoporous silica coating markedly enhanced the third-harmonic response of ZIF-8-based particles and reduced the cavitation threshold across the investigated size range, particularly in the low-intensity region. These findings support an interface-governed mechanism in which particle geometry and mesoporous shell structure modulate gas-nucleus stabilization and activation, providing a strategy for constructing MOF-based low-threshold cavitation nanoplatforms.

## Highlights

• ZIF-8-based nanostructures were engineered for cavitation enhancement.
• Bare ZIF-8 leads to a non-monotonic size-dependent apparent cavitation threshold.
• Particle morphology modulates cavitation in addition to material type.
• Mesoporous silica shells amplify low-threshold cavitation for all sizes.
• Interfacial engineering enables MOF-based low cavitation threshold.

## Graphical abstract

**Figure:**
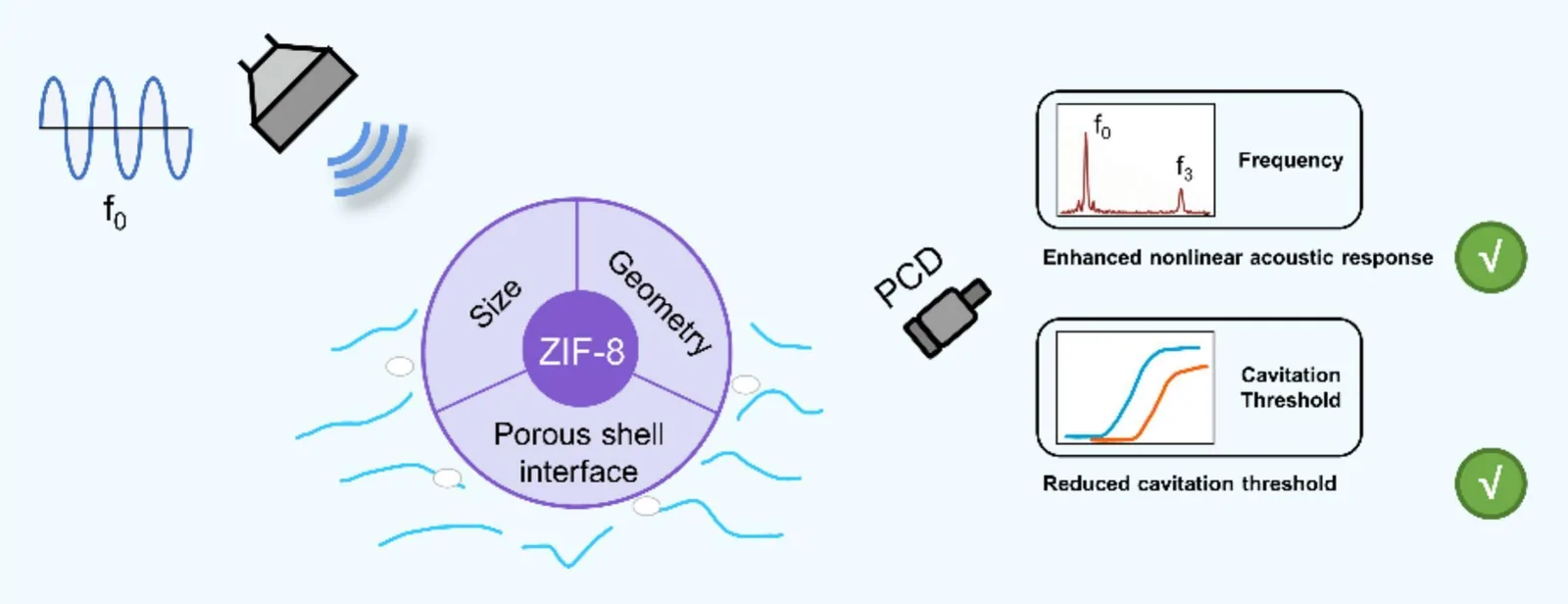

## 1 Introduction

Ultrasound-induced cavitation is the physical basis for many ultrasound-mediated therapeutic, drug delivery, and sonodynamic processes [1], [2], [3]. The oscillation, expansion, and collapse of cavitation bubbles [4], [5] in an acoustic field can induce localized mechanical perturbations, microjets, shear stress, and the generation of reactive oxygen species (ROS), thereby contributing to enhanced cell membrane permeabilization, drug release, tissue penetration, and sonodynamic therapy (SDT) [6], [7]. However, in practical biomedical applications, a key challenge is not simply whether cavitation can be generated, but whether it can be initiated and regulated under relatively low acoustic exposure. Lowering the acoustic threshold required to trigger cavitation-related responses may reduce the acoustic requirement and improve the controllability of ultrasound-mediated therapeutic and delivery processes [8], [9]. At the same time, low acoustic intensity should not be directly equated with biological safety when exogenous cavitation-regulating agents are present, because cavitation activity itself may introduce mechanical and vascular bioeffects that depend on the cavitation regime and not solely on the nominal acoustic exposure [10], [11]. Therefore, developing platforms that enable controllable cavitation under low-intensity ultrasound, together with appropriate evaluation of cavitation-related bioeffects, is of significant importance.

Gas-filled microbubbles are acoustically responsive, but owing to their micrometer-scale dimensions, they are generally confined to the vascular compartment [3], [12]. Phase-change nanodroplets offer smaller sizes and improved stability with greater potential for extravascular delivery, although acoustic droplet vaporization is required before strong bubble-mediated responses occur [13], [14]. In comparison, solid nanoparticle-based cavitation agents combine structural stability with nanoscale dimensions and offer greater flexibility in material and surface engineering [15], [16].

Nanoparticles provide important interfacial platforms for regulating ultrasound-induced cavitation [17], [18], [19]. In contrast to stochastic cavitation nucleation in homogeneous liquids, solid nano-interfaces can influence the formation, accommodation, and stabilization of incipient gas nuclei through surface defects, rough structures, pore environments, local wettability, and gas retention sites [15], [20]. Previous studies have shown that particle size [21], [22], surface morphology [23], [24], [25], porous structure [26], and interfacial chemistry [27], [28], [29] can all affect the cavitation threshold and nonlinear acoustic response. In particular, nanomaterials with porous structures or heterogeneous interfaces may provide more favorable environments for stabilizing incipient gas nuclei, thereby facilitating cavitation being triggered under low acoustic intensities. Current understanding of nanoparticle-regulated cavitation is derived from studies on silica nanoparticles, rough interfaces, or hydrophobically modified surfaces [16], [30], [31], [32], [33]. For metal–organic framework (MOF) materials [34], [35], especially zeolitic imidazolate framework-8 (ZIF-8) [36], their cavitation-related interfacial roles remain insufficiently investigated.

ZIF-8 is a representative MOF material, which attracted considerable attention in nanomedicine, including drug delivery, tumor microenvironment-responsive therapy, and sonodynamic therapy, owing to its tunable size and morphology, high specific surface area, well-defined microporous structure, readily engineerable surface, and pH-responsive degradability [37], [38], [39]. ZIF-8-based sonodynamic studies have mainly focused on ROS generation, Zn^2+^ release, drug loading, and enhanced therapeutic efficacy [40], [41], [42], whereas the interfacial physical role of ZIF-8 particles themselves in ultrasound-induced cavitation needs to be further investigated. In other words, whether ZIF-8 can serve as an effective cavitation-regulating interface, and how its structural parameters influence cavitation-triggering behavior, remain to be answered. In particular, how the particle size, external geometry, and pore structure of ZIF-8 systematically affect low-intensity cavitation, or whether the relatively limited intrinsic cavitation activity of ZIF-8 can be effectively enhanced through interfacial engineering. Establishing the relationships among ZIF-8 structural parameters, incipient gas nucleus stability, acoustic threshold, and nonlinear cavitation response is the key for understanding and designing MOF-based low-threshold cavitation platforms.

In this study, we used ZIF-8 as a model MOF nanoparticle and constructed a structurally tunable ZIF-8-based system, including bare ZIF-8 particles with different sizes, ZIF-8 particles with distinct external morphologies, and mesoporous silica-coated ZIF-8@SiO_2_ composite particles (Fig. 1a). By comparing their third-harmonic responses and apparent onset thresholds under low-intensity ultrasound, we systematically investigated the effects of particle size, external geometric boundaries, and mesoporous shell interfaces on cavitation-triggering capability. We propose that low-intensity cavitation in ZIF-8-based nanoparticles is not solely governed by material composition, but is closely associated with the ability of the particle interface to accommodate, stabilize, and activate incipient gas nuclei. This work aims to establish the relationship among structure, gas nucleus stability, acoustic threshold, and cavitation response, thereby providing an interfacial engineering basis for the design of MOF-based low-threshold cavitation nanoplatforms.

**Fig. 1:**
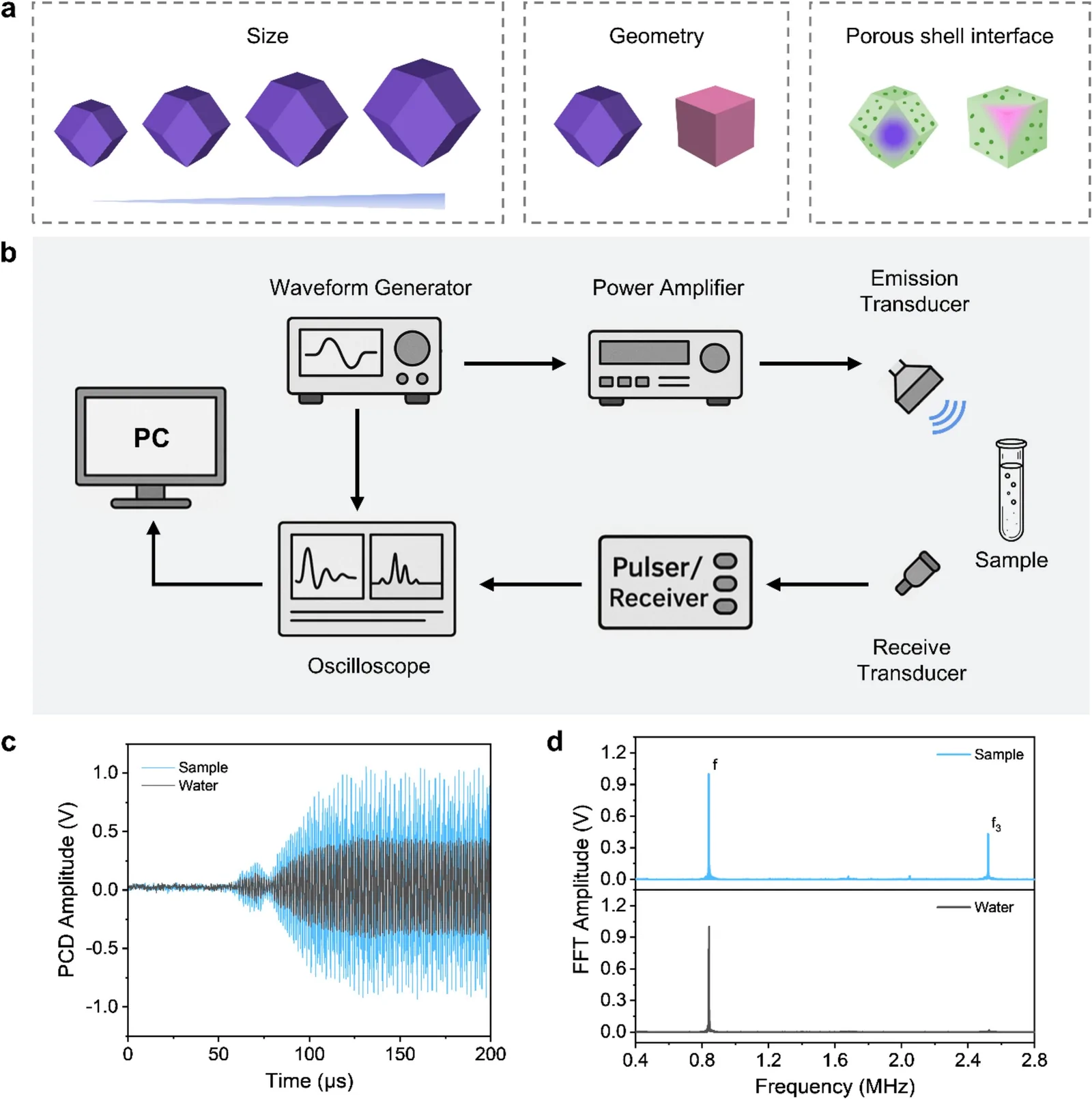
ZIF-8-based nanoparticle design and cavitation measurement. (a) Schematic illustration of the three investigated particle categories: size-varied ZIF-8, geometry-varied ZIF-8, and ZIF-8@SiO_2_. (b) Configuration of the PCD-based cavitation measurement system. (c) Representative time-domain PCD signals recorded during ultrasound irradiation. (d) Corresponding frequency spectra of the received PCD signals.

## 2 Materials and methods

### 2.1 Materials

Zinc nitrate hexahydrate (Zn(NO_3_)_2_·6H_2_O) was purchased from Aladdin Chemistry Co., Ltd. (China). 2-methylimidazole (2-MeIM) was obtained from Alfa Aesar Chemicals Co., Ltd. (China). Cetyltrimethylammonium bromide (CTAB) was supplied by Shanghai Macklin Biochemical Co., Ltd. (China). 8-arm polyethylene glycol (PEG, 10 kDa) was purchased from JenKem Technology Co., Ltd. (China). Tetraethyl orthosilicate (TEOS) was obtained from Sigma-Aldrich (China). Anhydrous methanol, anhydrous ethanol, and sodium chloride (NaCl) were purchased from Sinopharm Chemical Reagent Co., Ltd. (China). All chemicals were of analytical grade and used as received without further purification. Ultrapure water (18.2 MΩ·cm) from a Milli-Q water purification system was used in all experiments.

### 2.2 Synthesis of ZIF-8 NPs with different sizes

100 nm ZIF-8 NPs were synthesized according to a previously reported method with slight modifications [43]. Typically, 95.2 mg of Zn(NO_3_)_2_·6H_2_O was dissolved in 4 mL of methanol. Separately, 110.8 mg of 2-methylimidazole was dissolved in 4 mL of methanol and then poured into the above zinc nitrate solution. The mixture was stirred at room temperature for 2 h. The resulting product was collected by centrifugation, washed three times with methanol, and dried under vacuum at 60 °C.

For the preparation of 150, 200, and 250 nm ZIF-8 NPs, PEG was introduced as a mineralizer to regulate the particle size of ZIF-8 by lowering the nucleation energy barrier [44]. In brief, 20 mg of PEG was first added to the 2-methylimidazole aqueous solution, followed by the addition of the Zn(NO_3_)_2_·6H_2_O aqueous solution under stirring at room temperature. After 30 min of reaction, the products were collected by centrifugation, washed three times with ethanol and water, and dried under vacuum at 60 °C overnight. For 150 nm particles, 98.5 mg of 2-methylimidazole dissolved in 5.8 mL of water was mixed with 29.8 mg of Zn(NO_3_)_2_·6H_2_O dissolved in 2.2 mL of water. For 200 nm particles, 63.2 mg of 2-methylimidazole dissolved in 4.3 mL of water was mixed with 44.1 mg of Zn(NO_3_)_2_·6H_2_O dissolved in 3.7 mL of water. For 250 nm particles, 55.1 mg of 2-methylimidazole dissolved in 4.0 mL of water was mixed with 47.1 mg of Zn(NO_3_)_2_·6H_2_O dissolved in 4.0 mL of water.

### 2.3 Synthesis of cubic ZIF-8 NPs

CTAB was used as a morphology-directing agent to regulate the particle morphology [45]. Specifically, 454 mg of 2-methylimidazole dissolved in 6.6 mL of water was first mixed with 1.45 mg of CTAB dissolved in 0.4 mL of water and stirred for 5 min. Subsequently, 29.1 mg of Zn(NO_3_)_2_·6H_2_O dissolved in 1.0 mL of water was added, and the mixture was further stirred for 5 min. The reaction solution was then left undisturbed at room temperature for 3 h. The resulting product was collected by centrifugation, washed three times with ethanol, and dried under vacuum at 60 °C.

### 2.4 Synthesis of ZIF-8@SiO_2_ NPs

ZIF-8@SiO_2_ NPs were prepared using a modified procedure based on a previously reported method [46]. All of the above ZIF-8 NPs served as cores for SiO_2_ coating. In a typical synthesis, 25 mg of ZIF-8 NPs were dispersed in a mixed solution containing 15 mL of water, 10 mL of ethanol, 125 mg of CTAB, and 125 mg of 2-methylimidazole. After stirring for 15 min, 50 μL of TEOS was added dropwise into the mixture within 3 min. The reaction system was further stirred for 1 h. The final products were collected by centrifugation, washed with saturated NaCl/ethanol solution and water, and dried overnight under vacuum.

### 2.5 Characterizations

The morphology of the samples was investigated using scanning electron microscopy (SEM) on a Tescan MIRA4 and transmission electron microscopy (TEM) on a FEI Tecnai F20. Elemental mapping was performed using an Apollo XLT (EDAX, USA). The crystal structure of the samples was characterized by X-ray diffraction (XRD) using a Rigaku SmartLab SE diffractometer (Cu Kα radiation) with a scanning range of 5–45° and a scanning rate of 2°/min. Fourier transform infrared (FTIR) spectra were recorded on an IRspirit QATR-S spectrometer (Shimadzu, Japan). X-ray photoelectron spectra (XPS) were obtained using a Thermo Scientific K-Alpha spectrometer (USA). The specific surface area and pore size distribution of the samples were measured by nitrogen adsorption–desorption at 77 K using a Micromeritics ASAP2460 analyzer (USA). Dynamic light scattering (DLS) measurements were performed using a Malvern Zetasizer Nano ZS to analyze the particle size and zeta potential. Contact angle measurements were carried out using a Dataphysics OCA20 instrument.

### 2.6 Cavitation measurement and analysis

Fig. 1b shows the passive cavitation detection (PCD) system used in this study [47]. A sample chamber (40 mL), fabricated by 3D printing using acrylonitrile butadiene styrene (ABS), was placed in a water tank containing 80 L of degassed water. A home-designed 840 kHz transmitting transducer was calibrated using a needle hydrophone (NH0500, Precision Acoustics Ltd.). The acoustic pressure was measured at the sample position over the full range of driving voltages used in the cavitation experiments, and the resulting voltage–pressure calibration curve was used to determine the acoustic pressure corresponding to each driving condition. Representative time-domain pressure waveform and the calibration curve are provided in Fig. S1.

The transmitting transducer and a 5 MHz broadband receiving transducer (V309, Olympus) were arranged with their acoustic axes intersecting at the center of the sample chamber. The distances from the transmitting and receiving transducers to the chamber center were 45 and 37.5 mm, respectively, and this configuration was maintained throughout all measurements. Nanoparticle suspensions at a concentration of 0.5 mg/mL were used for the cavitation measurements. A sinusoidal excitation signal was generated by a waveform generator (Agilent 33522A) and amplified by a power amplifier (T&C Power Conversion AG1020) with a gain of 27 dB. Measurements were performed in tone-burst mode at 840 kHz with a burst length of 1000 cycles and a duty cycle of 50%. An acoustic absorber was positioned opposite the transmitting transducer to minimize acoustic reflections and standing-wave effects.

The cavitation signals were detected by the receiving transducer and amplified by a pulser/receiver (Olympus 5072PR) with a gain of 48 dB. After analog filtering with a 0.3 MHz high-pass filter and a 3 MHz low-pass filter, the signals were digitized using an oscilloscope (Tektronix TBS1000) at a sampling rate of 20 MHz under LabVIEW control (National Instruments). The receiving transducer was not calibrated for absolute acoustic-emission pressure, therefore, the PCD amplitudes were not interpreted as absolute cavitation-emission levels. Instead, all samples were evaluated using the same receiving geometry and identical amplification, filtering, and acquisition settings, and the resulting PCD amplitudes were used as normalized comparative responses [48]. For each nanoparticle formulation, three independently synthesized batches were used for acoustic measurements, with a separately prepared suspension from each batch measured under identical experimental conditions (n = 3).

The acquired raw signals were processed by fast Fourier transform (FFT), and the amplitude of the third harmonic (2.52 MHz) was extracted as a comparative indicator of nonlinear cavitation-related acoustic activity (Fig. 1c and d) [6], [49]. Under the present PCD configuration, the sub-harmonic component at 0.42 MHz remained weak and did not exhibit a sufficiently robust pressure-dependent response for reliable threshold determination. Because the measured amplitudes of spectral components at different frequencies are also influenced by the frequency-dependent response of the receiving system, the sub-harmonic and third-harmonic amplitudes were not interpreted as directly comparable absolute emission strengths [50]. Therefore, the third-harmonic response was used to derive the apparent onset threshold of the detectable nonlinear cavitation-related acoustic response. The pressure-dependent third-harmonic responses were fitted using a sigmoid function as Eq. (1). The tangent at the maximum-slope point of each fitted curve was then constructed, and its intersection with the pressure axis was defined as the apparent onset threshold.

(1) $$y=\frac{L}{1+e^{-k\left(x-x_{0}\right)}}$$

To complement the discrete harmonic analysis, broadband cavitation emissions were further evaluated for representative water, 150 nm ZIF-8, and 150 nm ZIF-8@SiO_2_ samples. For all samples and acoustic pressures, identical steady-state time-window lengths were selected after excluding the transient portions at the beginning and end of each waveform. The DC component was removed, followed by application of a Hann window, and the single-sided power spectral density (PSD) was calculated. Broadband analysis was performed over 0.50–2.80 MHz. Frequency intervals within ± 30 kHz of the fundamental, harmonic, and ultraharmonic components were excluded before integration of the remaining PSD [6], [51]. The resulting integrated value was defined as the relative broadband emission power. Because the PCD receiving system was not calibrated for absolute acoustic-emission pressure, the broadband values were used only for relative comparison under the identical measurement configuration.

For post-ultrasound stability evaluation, a suspension of representative 150 nm rhombic ZIF-8@SiO_2_ nanoparticles (0.5 mg/mL) was exposed to 840 kHz ultrasound at an acoustic pressure of 2.05 MPa for 60 s using 1000-cycle tone bursts at a duty cycle of 50%. Following ultrasound exposure, the particles were characterized by TEM and DLS.

## 3 Results and discussion

### 3.1 Material design and physicochemical characterization

A series of ZIF-8-based nanoparticles were constructed to systematically investigate the structural dependence of acoustic cavitation, including bare ZIF-8 particles with different sizes, bare ZIF-8 particles with distinct external geometries, and mesoporous silica-coated ZIF-8@SiO_2_ composite particles. Through this design, the effects of particle size, external geometric interface, and porous shell interface can be separately evaluated, thereby providing a model system for identifying the dominant structural factors responsible for variations in the cavitation threshold.

SEM images showed that the as-prepared bare ZIF-8 particles exhibited uniform and tunable size distributions (Fig. 2a–d). Rhombic dodecahedral ZIF-8 particles with sizes ranging from 100 to 250 nm were obtained under different synthetic conditions. Meanwhile, cubic ZIF-8 particles were successfully prepared by adjusting the synthesis parameters (Fig. 2e), providing a basis for comparing the effect of external geometry on cavitation behavior. TEM observations further confirmed the structural differences between bare ZIF-8 and ZIF-8@SiO_2_ particles (Figs. 2f–i and S2). Bare ZIF-8 particles showed relatively well-defined particle contours, whereas the ZIF-8@SiO_2_ samples exhibited distinctly roughened surfaces, with a mesoporous silica shell uniformly coated on the ZIF-8 core. The shell thickness was approximately 5 nm. The distributions of Zn, O, and Si in the elemental mapping images further verified the core–shell configuration, indicating that the mesoporous silica shell was successfully introduced onto the ZIF-8 surface (Fig. 2j–k). In addition, the zeta potential changed from positive to negative after coating, further supporting the successful formation of the silica layer (Fig. 3a). DLS results showed that the hydrodynamic diameters of the particles were consistent with TEM observations, and the polydispersity index (PDI) values were below 0.2, indicating uniform particle size distributions and good dispersibility (Fig. S3).

**Fig. 2:**
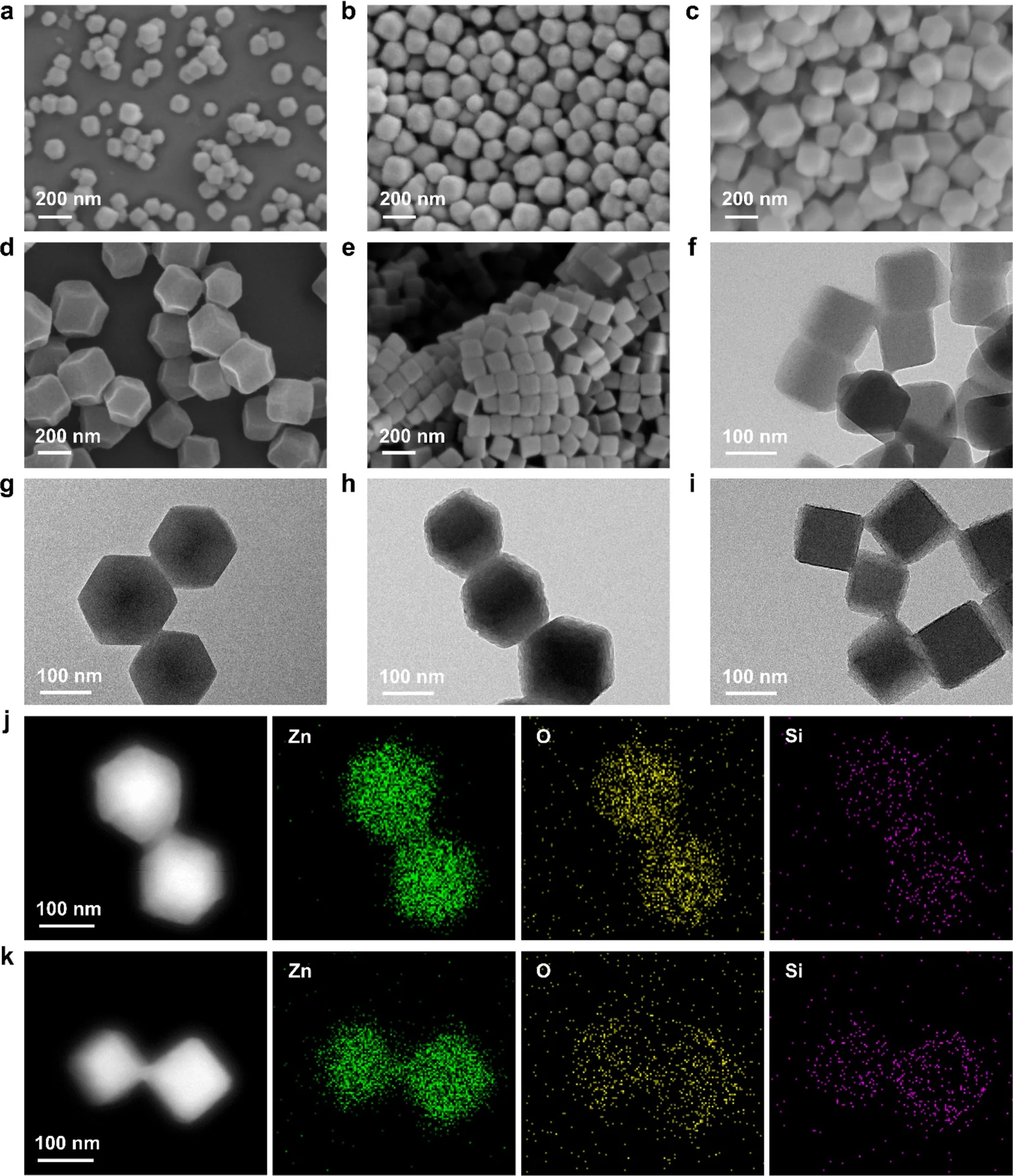
Morphological and compositional characterization of ZIF-8-based nanoparticles. (a–d) SEM images of rhombic dodecahedral ZIF-8 with sizes of 100, 150, 200, and 250 nm, respectively. (e) SEM image of cubic ZIF-8. (f, g) TEM images of cubic and rhombic dodecahedral ZIF-8. (h, i) TEM images of rhombic dodecahedral and cubic ZIF-8@SiO_2_. (j, k) Elemental mapping images of rhombic dodecahedral and cubic ZIF-8@SiO_2_.

**Fig. 3:**
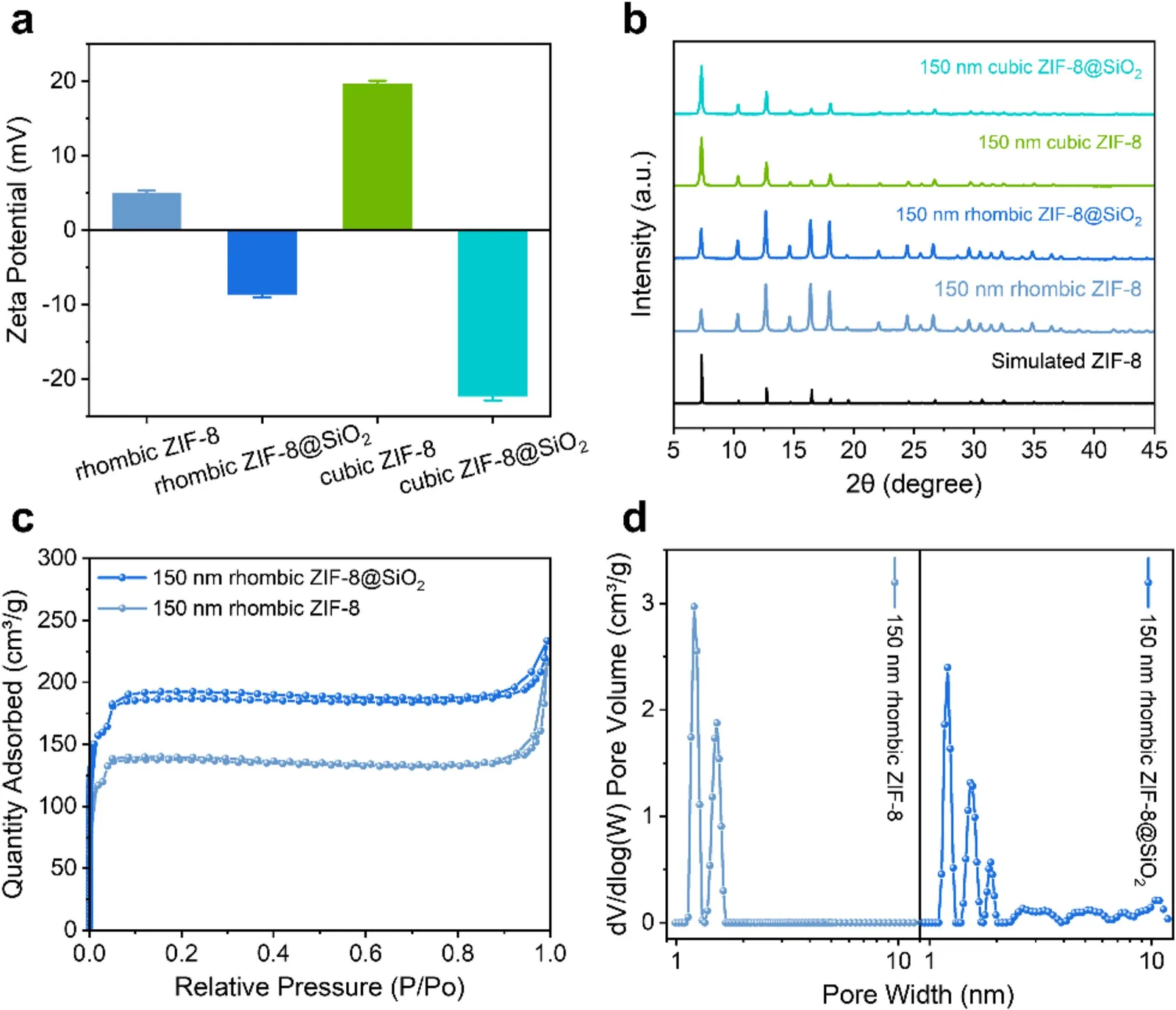
Structural and porous characteristics of representative bare and silica-coated ZIF-8 particles. (a) Zeta potential measurements. (b) XRD patterns. (c) N_2_ adsorption–desorption isotherms. (d) Pore size distributions.

XRD patterns showed that the bare ZIF-8 samples exhibited characteristic diffraction peaks of ZIF-8 with good crystallinity (Figs. 3b and S4). After coating, the characteristic diffraction peaks of ZIF-8 were still retained, indicating that the construction of the silica shell did not disrupt the main crystalline phase of the ZIF-8 core. Nitrogen adsorption–desorption isotherms and pore size distribution analyses further revealed the porous structural features of the samples (Figs. 3c–d and S5–6). Bare ZIF-8 mainly showed adsorption characteristics associated with its intrinsic microporous structure, with a type I isotherm and pore size distributions below 2 nm. In contrast, the ZIF-8@SiO_2_ samples exhibited typical type IV isotherms with a narrow and nearly parallel H4-type hysteresis loop, as indicated by the separation between the adsorption and desorption branches. This behavior is generally associated with bottleneck effects and ink-bottle-like pore structures, which may facilitate gas retention within the interconnected and tortuous pore network [52]. The pore size distribution confirmed the coexistence of micropores and mesopores in ZIF-8@SiO_2_, where the micropores originated from the ZIF-8 core, while the mesopores of approximately 3, 5, and 10 nm were mainly attributed to the silica shell. The specific surface area increased from 408.58 to 560.42 m^2^ g^−1^ after coating, indicating that the outer shell not only modified the external particle interface but also introduced additional porous contact interfaces.

FTIR (Fig. S7) and XPS (Fig. S8) analyses further confirmed differences in sample composition and interfacial states. In the FTIR spectra, ZIF-8 particles exhibited a characteristic C=N stretching vibration at 1584 cm^−1^, along with a series of bands in the range of 1350–1500 cm^−1^ associated with in-plane vibrations of the imidazole ring. In addition, the absorption bands at 1147 and 995 cm^−1^ were assigned to fingerprint out-of-plane bending vibrations of the imidazole ring. After silica coating, a strong and broad absorption band appeared at approximately 1080 cm^−1^, corresponding to the asymmetric stretching vibration of Si–O–Si bonds. Meanwhile, a broad absorption band near 3500 cm^−1^ was observed, which could be attributed to the stretching vibration of surface silanol groups on the SiO_2_ shell. XPS survey spectra showed that the binding energy positions and peak profiles of the main elements remained largely unchanged before and after coating, including Zn 2p (∼1045 and ∼1022 eV), O 1s (∼533 eV), N 1s (∼400 eV), and C 1s (∼285 eV), indicating that the main ZIF-8 framework was not significantly altered during silica coating. After SiO_2_ shell formation, new Si 2s and Si 2p signals appeared at ∼154 and ∼103 eV, respectively, further confirming the successful introduction of the SiO_2_ shell.

### 3.2 Size-dependent cavitation behavior of bare ZIF-8 nanoparticles

Unlike previous studies on the application of ZIF-8 in sonodynamic therapy, this work focuses on its physical role as a cavitation-related interface. The nonlinear acoustic responses of bare ZIF-8 nanoparticles with different particle sizes under low-intensity ultrasound were compared to establish the baseline cavitation behavior of the unmodified ZIF-8 system.

Acoustic measurements showed that bare ZIF-8 particles were not particularly strong cavitation enhancers overall, but their cavitation response exhibited a clear particle-size dependence (Fig. 4a). As the acoustic pressure increased, all samples with different particle sizes displayed gradually enhanced third-harmonic responses, indicating that bare ZIF-8 could participate in nonlinear acoustic processes associated with stable cavitation under certain conditions. However, the onset of the response varied among samples with different particle sizes. When the third-harmonic signal was used as the criterion for cavitation onset, the intermediate-sized sample (150 nm) generated a detectable nonlinear response at a relatively low acoustic intensity, whereas smaller and larger particles required relatively higher excitation intensities.

**Fig. 4:**
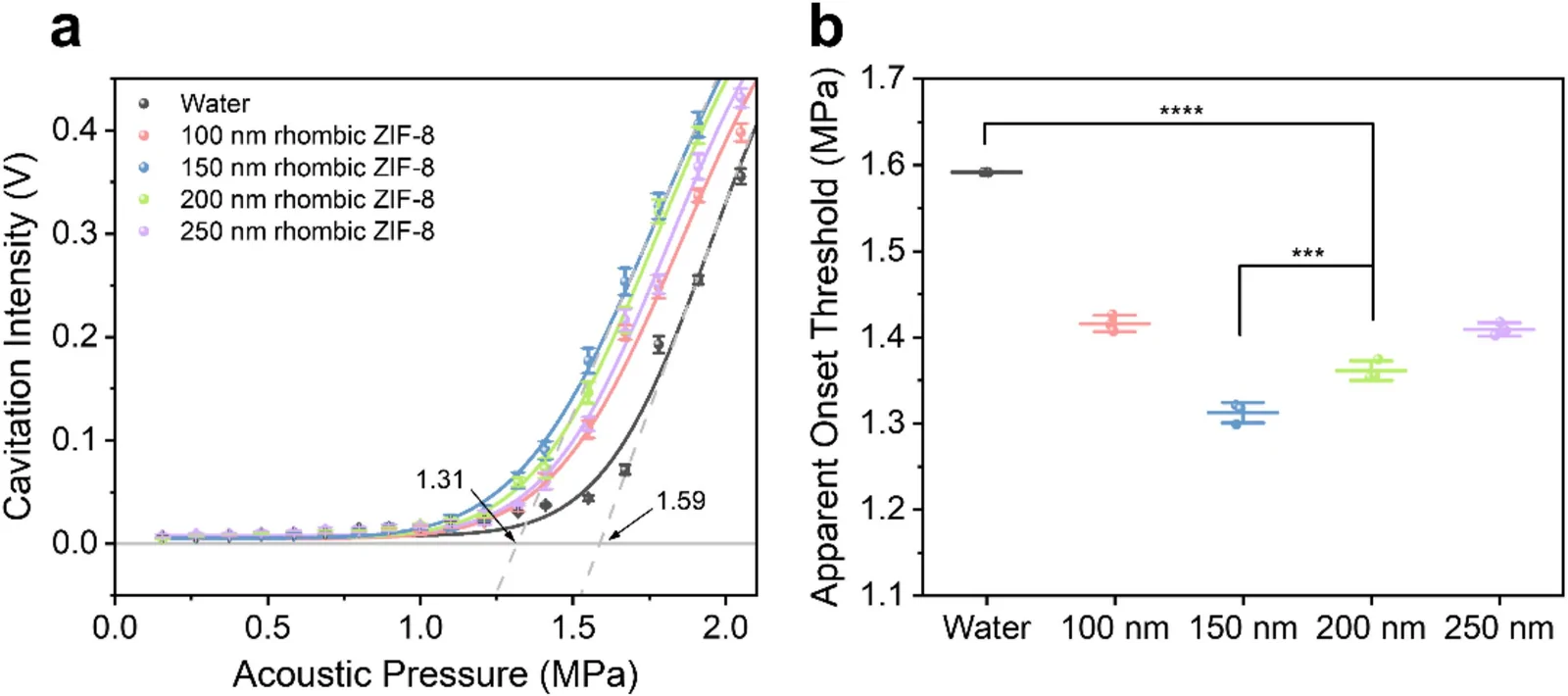
Size-dependent cavitation behavior of rhombic dodecahedral ZIF-8 particles. (a) Acoustic pressure–intensity curves of ZIF-8 particles with different sizes, fitted using a sigmoid function. The intensity is presented as the normalized third-harmonic amplitude. (b) Statistical comparison of the apparent onset thresholds for ZIF-8 particles with different sizes. Data are presented as mean ± SD from three independently synthesized batches (n = 3). Statistical significance was analyzed by one-way ANOVA (***P < 0.001, ****p < 0.0001).

Further threshold analysis confirmed that the apparent onset threshold of bare ZIF-8 did not vary monotonically with increasing or decreasing particle size, but instead exhibited a clear non-monotonic trend (Fig. 4b). Specifically, the 150 nm sample showed the lowest threshold (1.31 MPa), whereas both smaller and larger particles exhibited higher thresholds, although all values remained lower than that of pure water (1.59 MPa). This result indicates that, in this system, particle size does not influence cavitation in a simple monotonic manner, but is associated with multiple competing interfacial structural factors.

A plausible explanation is that, as particle size decreases, the increased external curvature may be unfavorable for the stable accommodation of incipient gas nuclei at the particle–liquid interface [53], thereby requiring a higher acoustic pressure to trigger a detectable cavitation response. Conversely, as particle size increases, the lower interfacial curvature may be more favorable for gas nucleus accommodation, but at the same mass concentration, the particle number decreases, which may reduce the total number of effective interfacial events available for triggering cavitation [54]. Therefore, the minimum threshold is likely to correspond to a balance between gas nucleus stability and the probability of effective nucleation events.

It should be noted that this optimal particle size does not imply that bare ZIF-8 has already formed a highly optimized cavitation-active interface. Rather, it should be understood as a constrained baseline state of the bare ZIF-8 system, in which particle size can only partially improve gas nucleus accommodation and cavitation-triggering conditions in the absence of additional interfacial engineering.

### 3.3 Geometry-dependent cavitation behavior of ZIF-8 nanoparticles

If cavitation behavior is indeed affected by interfacial geometric factors, then the external morphology of the particles, in addition to particle size, should also contribute to the regulation of their acoustic response. Based on this consideration, the cavitation behavior of ZIF-8 particles with different morphologies was further compared. Two ZIF-8 particles with distinct crystal shapes, namely rhombic dodecahedral and cubic ZIF-8, were selected to examine the independent contribution of geometric boundaries to cavitation behavior.

Under identical testing conditions, rhombic dodecahedral and cubic ZIF-8 samples with the same particle size of 150 nm exhibited distinguishable nonlinear acoustic responses (Fig. 5a). Although both samples were bare ZIF-8 with the same chemical composition, their third-harmonic response intensities and apparent onset thresholds were different, with the rhombic dodecahedral sample reaching the threshold at a lower acoustic pressure (Fig. 5b), which prove that external geometric features of nanoparticles indeed influence cavitation initiation.

**Fig. 5:**
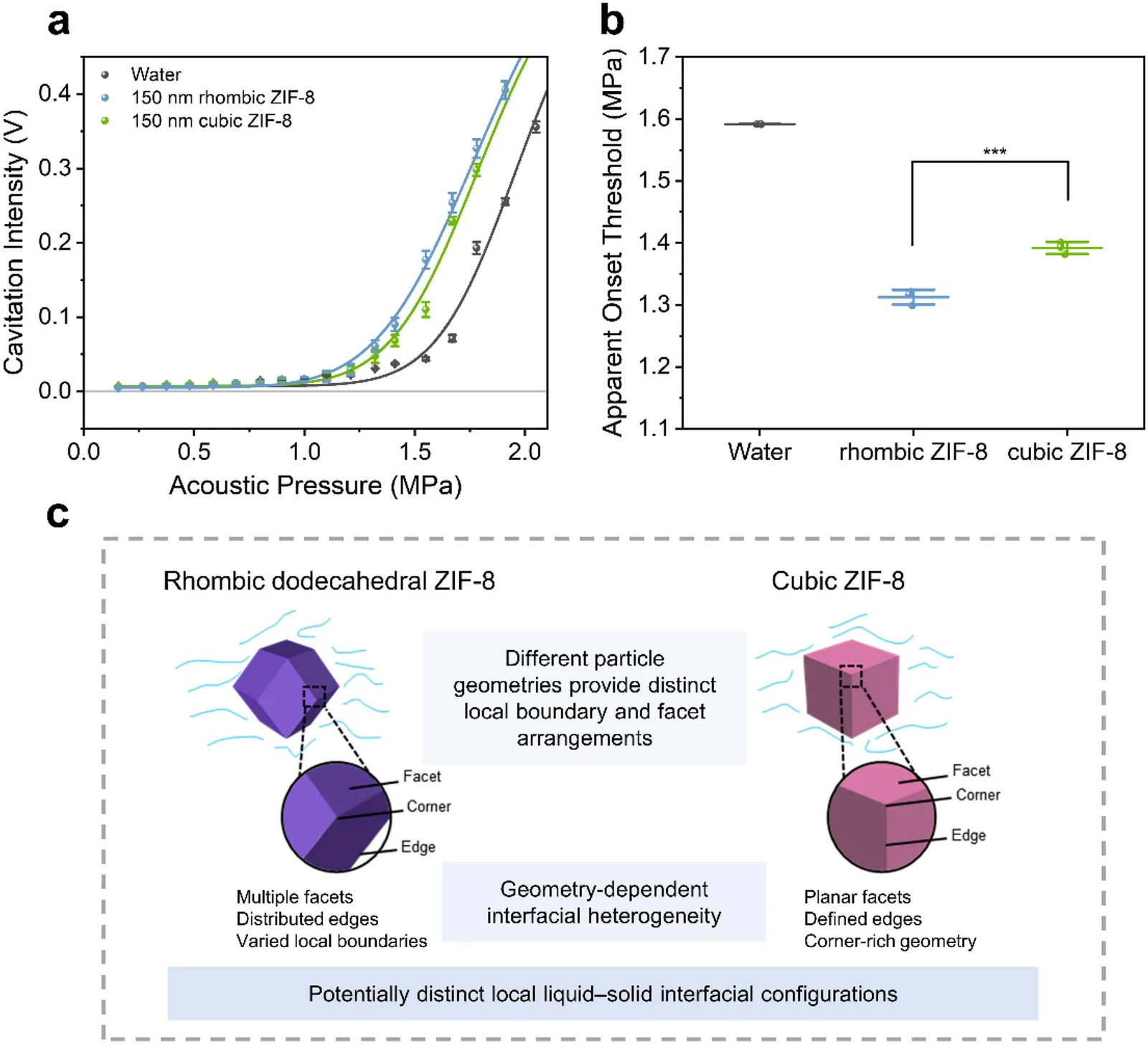
Geometry-dependent cavitation behavior of ZIF-8 particles. (a) Acoustic pressure–intensity curves of ZIF-8 particles with different geometries, fitted using a sigmoid function. The intensity is presented as the normalized third-harmonic amplitude. (b) Statistical comparison of the apparent onset thresholds. (c) Simplified schematic illustration of the geometric features of rhombic dodecahedral and cubic ZIF-8 particles. Data are presented as mean ± SD from three independently synthesized batches (n = 3). Statistical significance was analyzed by one-way ANOVA (***P < 0.001).

This result indicates that cavitation behavior in the ZIF-8 system is not determined solely by material composition, specific surface area, or microporous structure; the external geometric interface also contributes to the establishment of cavitation-initiation conditions. Rhombic dodecahedral ZIF-8 exposes 12 {110} facets, providing a larger number of faces, edges, and corner features, whereas the growth of cubic ZIF-8 is mainly limited by the {100} facets [55], resulting in six mutually perpendicular faces with equal edge lengths and regular corners close to 90°. Different combinations of exposed crystal facets, edge–corner characteristics, and local interfacial heterogeneity may collectively affect the contact state of liquid at the particle surface and the stability of potential gas nuclei (Fig. 5c).

### 3.4 Mesoporous silica shell engineering enhances low-intensity cavitation

Although both particle size and morphology could regulate the cavitation behavior of bare ZIF-8, the overall enhancement remained limited. This suggests that the ZIF-8 framework itself may not serve as an intrinsically efficient cavitation interface, and that further interfacial engineering is required to amplify its low-intensity cavitation-triggering capability. Based on the above results, it was hypothesized that the key factor governing ZIF-8 cavitation behavior is not only its intrinsic material properties, but also whether it can provide a favorable interface for gas nucleus formation and stabilization. To test this hypothesis, ZIF-8@SiO_2_ composite particles were further constructed, in which a mesoporous silica shell was introduced to reconstruct the external interface.

Compared with bare ZIF-8, the ZIF-8@SiO_2_ composite particles exhibited markedly enhanced third-harmonic responses under the same ultrasound conditions (Fig. 6a and b). In particular, in the lower acoustic intensity range, the coated samples were already able to generate detectable and stronger nonlinear acoustic signals, whereas the corresponding bare ZIF-8 samples still showed weak or negligible responses. This result indicates that the introduction of a mesoporous silica shell enables ZIF-8-based particles to trigger stable cavitation-related responses under milder acoustic conditions.

**Fig. 6:**
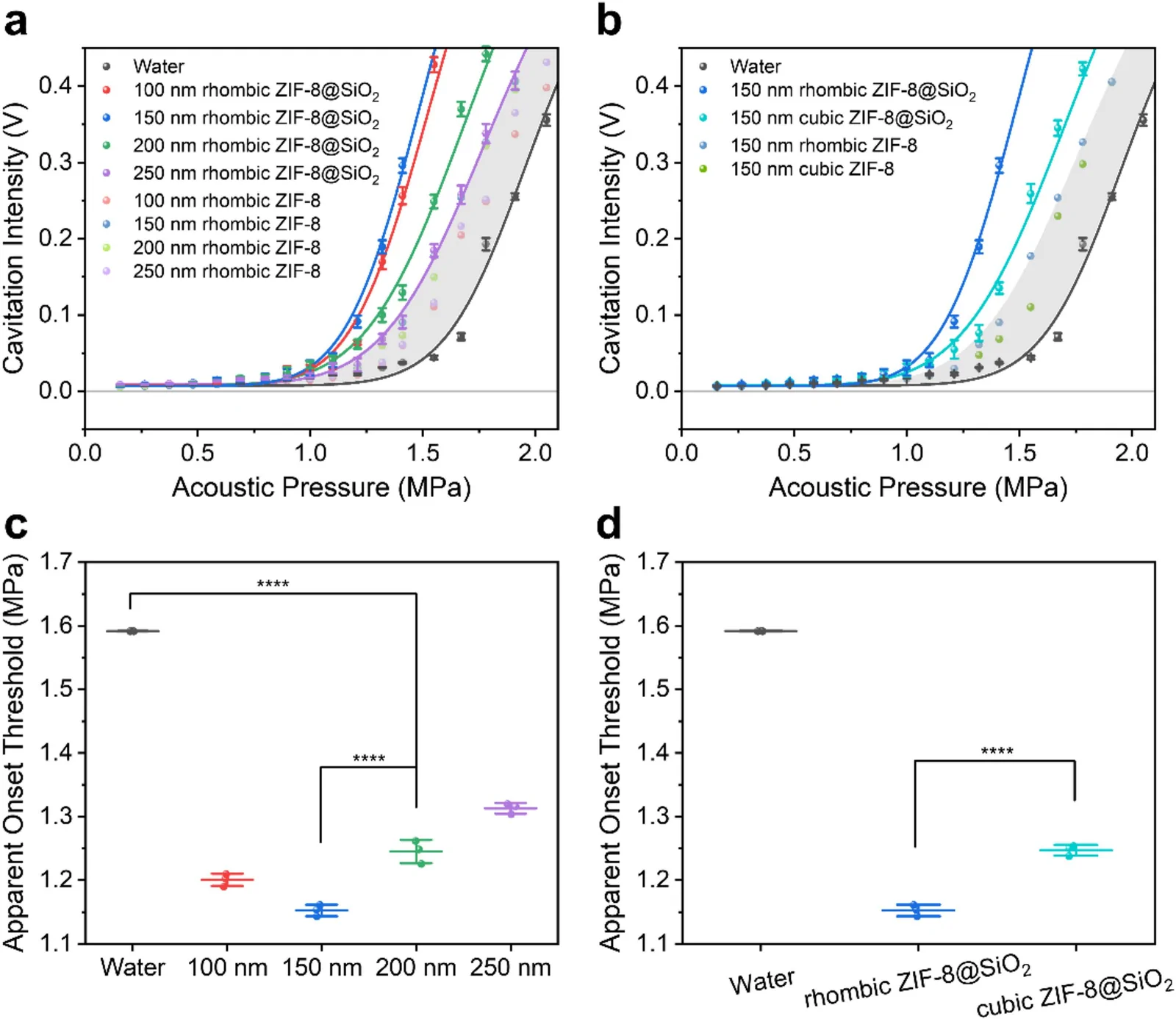
Sigmoid-fitted acoustic pressure–intensity curves based on normalized third-harmonic amplitude for (a) silica-coated rhombic dodecahedral ZIF-8 with different sizes and (b) silica-coated ZIF-8 with different geometries. Corresponding apparent onset thresholds for (c) size-varied and (d) geometry-varied silica-coated ZIF-8 particles. Data are presented as mean ± SD from three independently synthesized batches (n = 3). Statistical significance was analyzed by one-way ANOVA (****p < 0.0001).

To determine whether the enhanced acoustic response extended beyond discrete harmonic emissions, broadband cavitation-related activity was further examined for representative 150 nm ZIF-8, and 150 nm ZIF-8@SiO_2_ samples water (Fig. S9). The broadband emission from water increased progressively with acoustic pressure, whereas both nanoparticle-containing suspensions exhibited a pronounced increase at lower acoustic pressures. This trend was evident in both the representative frequency spectra and the relative broadband emission power, indicating that the nanoparticles facilitate the development of broadband cavitation-related acoustic activity under relatively low acoustic exposure. Broadband emissions are commonly associated with transient or collapse-dominated cavitation dynamics [56]. However, quantitative determination of an inertial-cavitation onset threshold requires a specific criterion for the broadband response. Therefore, the broadband analysis was used here as complementary characterization rather than to define a separate inertial-cavitation threshold. What we are interested here is non-inertial cavitation, the threshold refers to the bubble formation threshold. When there are bubbles produced in water, the medium becomes strongly non-linear, hence third harmonic signals is a good indicator for the presence of bubbles in water.

Threshold analysis further showed that mesoporous silica coating significantly reduced the acoustic intensity required for cavitation triggering (Fig. 6c and d). Compared with bare ZIF-8, the apparent onset thresholds of the ZIF-8@SiO_2_ samples decreased overall. This result indicates that the introduction of the shell did not merely change the apparent composition of the particles, but effectively altered the particle–liquid contact interface, making it easier to trigger cavitation-related nonlinear oscillations under low-intensity ultrasound.

Notably, mesoporous silica coating did not enhance cavitation only for a specific particle size, but produced a broadly improved cavitation response across the investigated size range. This result indicates that the porous interface and surface heterogeneity introduced by the silica shell provide a generally favorable interfacial gain, improving the cavitation-triggering conditions on ZIF-8 cores of different sizes under low-intensity ultrasound. However, the optimal size trend was still preserved after coating, suggesting that the baseline geometric constraint imposed by particle size was not eliminated. Instead, it was superimposed with shell-mediated interfacial engineering to jointly regulate the cavitation behavior. Therefore, compared with particle-size tuning alone, interfacial engineering represents a more effective strategy for improving the low-threshold cavitation capability of ZIF-8-based nanoparticles.

This enhancement may be attributed to the reconstruction of the local interface by the mesoporous silica shell. On one hand, the mesoporous shell provides abundant multiscale pore structures as well as rough and heterogeneous interfaces, which may increase the probability of local gas retention, gas enrichment, and incipient gas nucleus survival [57]. On the other hand, the shell transforms the external surface of the particles from exposed ZIF-8 crystal facets into a more complex porous SiO_2_ contact interface, thereby increasing the number of potential nucleation sites and improving gas nucleus stability under the acoustic field [58]. Therefore, the enhanced cavitation performance of ZIF-8@SiO_2_ should not be interpreted as a simple additive effect of two materials, but rather as an interfacial amplification effect. The mesoporous silica shell should thus be regarded not as a passive coating, but as an active interfacial amplification layer for low-intensity cavitation.

Although contact angle (CA) measurements showed that all samples generally exhibited hydrophilic surfaces (Fig. S10), their cavitation behaviors differed markedly, indicating that wettability alone is insufficient to explain the threshold variations in this system. Instead, nanoscale geometric features and porous interfacial structures are more likely to play dominant roles.

To evaluate the structural stability of the mesoporous silica shell under the highest acoustic pressure used in the cavitation measurements, representative 150 nm rhombic ZIF-8@SiO_2_ nanoparticles were exposed to 840 kHz ultrasound at 2.05 MPa for 60 s using 1000-cycle tone bursts at a duty cycle of 50%. Compared with the morphology before ultrasound exposure (Fig. 2h), the post-ultrasound TEM image showed that the particles retained their overall rhombic core–shell morphology, without obvious disruption of the silica shell, particle fragmentation, or structural collapse (Fig. S11a). Moreover, the post-ultrasound DLS size distribution remained comparable to the initial distribution shown in Fig. S3f, with no substantial shift or broadening and no evidence of pronounced aggregation (Fig. S11b). These observations support the structural integrity and colloidal stability of the representative ZIF-8@SiO_2_ nanoparticles under the investigated ultrasound conditions.

Taken together, the mesoporous silica shell substantially enhanced the cavitation response of ZIF-8-based particles under low-intensity ultrasound. This indicates that the optimization of cavitation behavior does not depend solely on the intrinsic material properties of ZIF-8, but is governed by the collective regulation of the gas nucleus interface by multiple structural factors.

### 3.5 Quantitative comparison and proposed mechanism

To quantitatively compare the relative contributions of different structural factors, threshold reduction ratios were calculated using pure water, cubic ZIF-8, or rhombic dodecahedral ZIF-8 as the corresponding reference. R_water_, R_geo_, and R_shell_ were used to describe the threshold reduction relative to pure water, the geometry-induced threshold reduction, and the shell-induced threshold reduction, respectively (Eq. (2), (3), (4)).

(2) $$R_{\mathrm{water}}=\left(P_{\mathrm{water}}-P_{\mathrm{sample}}\right)/P_{\mathrm{water}}\times 100\%$$

(3) $$R_{\mathrm{geo}}=\left(P_{\mathrm{cube}}-P_{\mathrm{dodeca}}\right)/P_{\mathrm{cube}}\times 100\%$$

(4) $$R_{\mathrm{shell}}=\left(P_{\mathrm{bare}}-P_{\mathrm{coated}}\right)/P_{\mathrm{bare}}\times 100\%$$

As shown in Table 1, the representative quantitative comparison indicates that different structural factors contributed to threshold reduction in distinct ways. The size-optimized bare ZIF-8 sample showed a 17.6% threshold reduction relative to pure water, suggesting that particle size defines an important baseline effect in the unmodified ZIF-8 system. Mesoporous silica coating further reduced the threshold by 12.9% relative to the corresponding bare ZIF-8 particles, indicating a strong additional interfacial amplification effect after particle construction. In comparison, the geometry-induced threshold reduction was more moderate, with rhombic dodecahedral ZIF-8 showing a 5.8% lower threshold than cubic ZIF-8. Although smaller in magnitude, this difference still confirms that external geometric boundaries participate in cavitation regulation. These quantitative differences indicate that threshold reduction cannot be attributed to a single structural descriptor. Instead, particle size, geometry, and shell engineering may regulate cavitation by modifying the energetic and probabilistic conditions for incipient gas nucleus accommodation.

**Table 1:** Representative quantitative comparison of structural contributions to apparent onset threshold reduction.

Structural factor	Representative comparison	Quantitative descriptor	Mechanistic implication
Size	150 nm ZIF-8 vs water	R_water_=17.6%	Intermediate size gives the best balance between gas nucleus stability and nucleation probability
Geometry	Rhombic dodecahedral vs cubic ZIF-8	R_geo_=5.8%	External facets, edges, and corners modulate local nucleation conditions
Shell	150 nm ZIF-8@mSiO_2_ vs 150 nm ZIF-8	R_shell_=12.9%	Mesoporous shell provides interfacial amplification
Wettability	Contact angle of bare ZIF-8 vs ZIF-8@mSiO_2_	All samples remain hydrophilic	Macroscopic wettability alone is insufficient
Porosity	BET surface area and pore size of bare ZIF-8 vs ZIF-8@mSiO_2_	S_BET_: 408.58 → 560.42 m^2^ g^−1^; mesopores: ∼3, ∼5, ∼10 nm	Mesopores and ink-bottle-like pores may favor gas retention

From the viewpoint of interfacial energy, Young’s equation (Eq. (5)) describes the relationship among solid–vapor, solid–liquid, and liquid–vapor interfacial tensions and the macroscopic contact angle [14]. This relationship indicates that wettability is closely associated with the interfacial energy state. However, in the present system, all samples remained hydrophilic while showing clearly different thresholds, suggesting that macroscopic wettability alone cannot explain the observed threshold variations.

(5) $$\gamma_{\mathrm{SV}}-\gamma_{\mathrm{SL}}=\gamma_{\mathrm{LV}}\cos\theta$$

The stability of incipient gas nuclei can be further considered using the Laplace pressure relationship (Eq. (6)), where r is the gas nucleus radius [4]. A smaller gas nucleus requires a higher internal pressure and is therefore less stable [44], [45]. This provides a physical basis for the non-monotonic size dependence of bare ZIF-8: highly curved small particles may be unfavorable for stable gas nucleus accommodation, whereas larger particles provide fewer effective interfacial events at the same mass concentration. Meanwhile, mesoporous or concave interfacial structures introduced by the silica shell may facilitate gas retention and nucleus survival.

(6) $$\Delta P=2\gamma_{\text{LV}}/r$$

In classical heterogeneous nucleation, the nucleation barrier at a solid interface can be expressed as a fraction of the homogeneous nucleation barrier (Eq. (7)). Although f(θ) is related to wettability, in nanoscale porous and rough interfaces, the effective barrier is also influenced by pore geometry, curvature, edge–corner features, and gas retention sites [27], [47], [48]. This explains why samples with similar macroscopic hydrophilicity can still show different cavitation thresholds.

(7) $$\Delta G_{\text{hetero}}^{*}=f\left(\theta \right)\Delta G_{\text{homo}}^{*}$$

Based on the above quantitative comparison and interfacial-energy analysis, the cavitation behavior of ZIF-8-based nanoparticles should be understood as combined outcome of particle size, geometric boundaries, and porous shell interfaces. We propose a working model to explain the cavitation behavior of ZIF-8-based nanoparticles (Fig. 7). This model is established based on the correlation between structural differences and acoustic responses, and is intended to provide a rational framework for the design of more effective nanoparticles, rather than to offer a definitive description of the microscopic nucleation process. Within this model, the ability of the particle interface to accommodate, stabilize, and activate incipient gas nuclei is considered the key factor governing the threshold under low-intensity ultrasound.

**Fig. 7:**
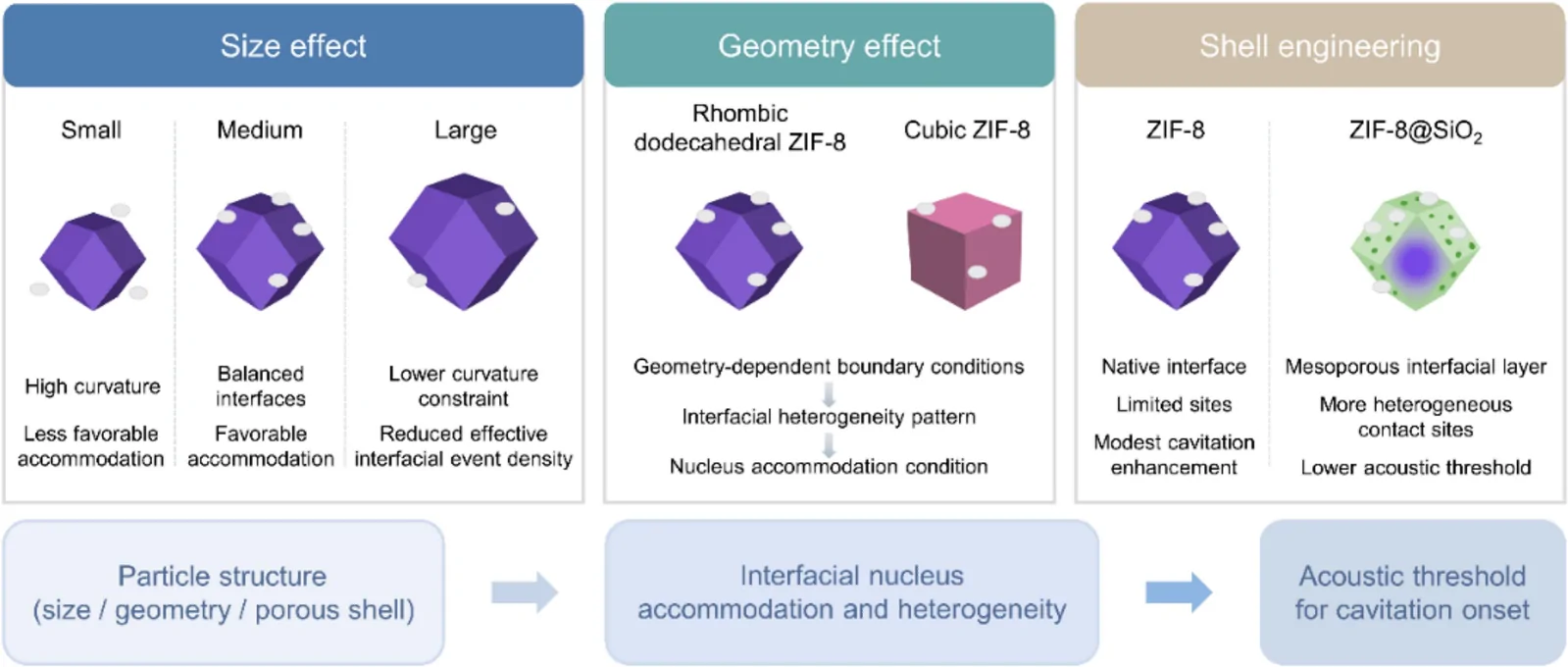
Proposed mechanism for cavitation regulation in ZIF-8-based nanostructures.

For bare ZIF-8 nanoparticles, the non-monotonic size dependence of the apparent onset threshold indicates that particle size affects results from at least two simultaneous processes. On one hand, smaller particles possess higher surface curvature, which is unfavorable for stabilizing local gas nuclei. On the other hand, although larger particles may be geometrically more favorable for gas nucleus stabilization, the number of effective particles available at a given concentration decreases, thereby limiting the overall probability of gas bubble nucleation. Therefore, the optimal particle size results from a competitive balance between gas nucleus stability and event probability of effective nucleation.

Within this framework, the morphology effect can be understood as a further modulation of interfacial conditions by the external geometric boundary. Edge features, combinations of exposed crystal facets, and local interfacial heterogeneity associated with different crystal shapes may alter the contact state of liquid on the particle surface and influence the spatial distribution of local nucleation events. In other words, morphological differences do not necessarily provide the strongest cavitation enhancement by themselves, they tell us that cavitation behavior is not determined solely by the chemical composition or microporous structure of ZIF-8; the external geometric interface also contributes to the establishment of cavitation-triggering conditions.

By contrast, the role of the mesoporous silica shell is more directly reflected in interfacial reconstruction. The SiO_2_ shell introduces multiscale pore structures, rough interfaces, and surface heterogeneity, which may increase the probability of local gas retention, gas enrichment, and incipient gas nucleus survival. Meanwhile, after coating, the external surface of the particles is no longer composed of exposed bare ZIF-8 crystal facets alone, but is transformed into a more complex porous SiO_2_ contact interface, thereby increasing the number of potential nucleation sites and improving cavitation-triggering conditions under low-intensity ultrasound. Therefore, the marked enhancement observed for ZIF-8@SiO_2_ is not merely due to the addition of another material layer, but represents a transition from a material-intrinsic effect to an interface-engineering effect.

It can therefore be inferred that particle size, geometry, and the mesoporous shell are not isolated factors, but regulate the relationship between the particle interface and gas nuclei at different levels. Particle size defines the baseline geometric constraint in the bare ZIF-8 system, morphology further modifies the local interfacial boundary conditions, and the mesoporous silica shell markedly amplifies gas nucleus accommodation and cavitation triggering by constructing a porous and heterogeneous interface. These factors converge on the same mechanistic core: the key to low-intensity cavitation is not whether the particle possesses a specific material composition, but whether its interface can effectively stabilize and activate incipient gas nuclei.

Therefore, this study establishes a “structure–gas nucleus stability–acoustic threshold” mechanistic framework. For MOF-based cavitation nanoplatforms, future design should not rely solely on the intrinsic material properties, specific surface area, or particle-size regulation, but should place greater emphasis on engineering the external particle interface. By tuning particle size, external geometry, and porous shell structure, the gas nucleus accommodation environment can be more effectively optimized, the cavitation threshold can be reduced, and the controllability of cavitation triggering under low-intensity ultrasound can be improved. This understanding not only helps clarify the cavitation behavior of ZIF-8-based systems, but also provides a generalizable interfacial engineering principle for the low-threshold cavitation design of other MOFs or porous nanoparticles.

## 4 Conclusion

In this study, we systematically investigated the cavitation behavior of ZIF-8-based nanoparticles under low-intensity ultrasound, with particular emphasis on the effects of particle size, external morphology, and mesoporous silica shell engineering on the cavitation threshold and nonlinear acoustic response. The results showed that bare ZIF-8 particles are not intrinsically strong cavitation enhancers overall; nevertheless, their cavitation behavior exhibited a clear size dependence, with the lowest threshold observed in the intermediate size range. This non-monotonic trend suggests that low-intensity cavitation is not simply determined by particle size alone, but is likely governed by a competitive balance among interfacial curvature, gas nucleus stability, and the probability of effective nucleation events. The comparison between ZIF-8 particles with different morphologies further indicates that the external geometric boundary of the particles also contributes to the establishment of cavitation-triggering conditions.

More importantly, the mesoporous silica shell markedly enhanced the nonlinear acoustic response of ZIF-8-based particles under low-intensity ultrasound and reduced the cavitation threshold. This enhancement indicates that the SiO_2_ shell is not merely a passive coating, but rather serves as an active interfacial amplification layer that improves incipient gas nucleus accommodation and cavitation-triggering conditions by introducing porous interfaces and surface heterogeneity. Based on these findings, this study proposes a “structure–gas nucleus stability–acoustic threshold” mechanistic framework, emphasizing that the design of MOF-based low-threshold cavitation platforms should prioritize interfacial engineering rather than relying solely on material composition, particle size, or specific surface area. This understanding provides guidance for the rational design of ZIF-8 and other porous nanoparticles for low-intensity ultrasound therapy, drug delivery, and sonodynamic-related applications.

## CRediT authorship contribution statement

**Xiaoman Zhang:** Writing – original draft, Investigation. **Jinyu Lin:** Methodology, Formal analysis, Data curation. **Qi Zhou:** Validation, Supervision, Resources. **Wenwu Cao:** Writing – review & editing, Project administration, Conceptualization.

## Declaration of competing interest

The authors declare that they have no known competing financial interests or personal relationships that could have appeared to influence the work reported in this paper.

## Appendix A Supplementary material

Supplementary Data 1 The Supplementary material contains Figures S1–S11, providing additional data supporting the results presented in the main text.
