# Supplementary material for: Interfacial regulation of low-intensity acoustic cavitation by MOF-based nanoparticles
Source: Ultrason Sonochem. 2026 Sep 27;134:108064. doi: 10.1016/j.ultsonch.2026.108064 (PMC13654563; doi:10.1016/j.ultsonch.2026.108064)
Supplement: Supplementary file 1 [file mmc1.docx]

Supplementary Information for

**Interfacial Regulation of Low-Intensity Acoustic Cavitation by MOF-Based Nanoparticles**

Xiaoman Zhang ^#^, Jinyu Lin ^#^, Qi Zhou ^*^, Wenwu Cao *

Center of Acoustic Functional Materials and Applications, School of Advanced Manufacturing Engineering, Nanjing University, Suzhou 215163, China

^#^ These authors contributed equally to this work.

^*^Corresponding Authors emails: [wcao@nju.edu.cn](mailto:wcao@nju.edu.cn) (Wenwu Cao), [iquohz@nju.edu.cn](mailto:iquohz@nju.edu.cn) (Qi Zhou).

**Fig. S1.** Acoustic pressure calibration of the 840-kHz transmitting transducer. (a) Representative time-domain acoustic pressure waveform. (b) Relationship between the applied driving voltage amplitude and the measured acoustic pressure at the sample position.

**Fig. S2.** TEM images of bare and SiO_2_-coated rhombic dodecahedral ZIF-8. (a–c) ZIF-8 particles of 100, 200, and 250 nm. (d–f) ZIF-8@SiO_2_ particles of 100, 200, and 250 nm.

**Fig. S3.** DLS size distributions of bare and SiO₂-coated ZIF-8 particles. (a–d) Rhombic dodecahedral ZIF-8 particles of 100, 150, 200, and 250 nm. (e–h) Rhombic dodecahedral ZIF-8@SiO_2_ particles of 100, 150, 200, and 250 nm. (i, j) Cubic ZIF-8 and ZIF-8@SiO_2_ particles.

**Fig. S4.** XRD patterns of 100, 200, and 250 nm rhombic dodecahedral ZIF-8 and corresponding ZIF-8@SiO_2_ particles.

**Fig. S5.** N_2_ adsorption–desorption isotherms of bare and SiO_2_-coated ZIF-8 particles. (a) Rhombic dodecahedral ZIF-8 particles of 100, 200, and 250 nm. (b) Rhombic dodecahedral ZIF-8@SiO_2_ particles of 100, 200, and 250 nm. (c) Cubic ZIF-8 and ZIF-8@SiO_2_ particles.

**Fig. S6.** Pore size distributions of bare and SiO_2_-coated ZIF-8 particles. (a) Rhombic dodecahedral ZIF-8 particles of 100, 200, and 250 nm. (b) Rhombic dodecahedral ZIF-8@SiO_2_ particles of 100, 200, and 250 nm. (c) Cubic ZIF-8 and ZIF-8@SiO_2_ particles.

**Fig. S7.** FTIR spectra of bare and SiO_2_-coated ZIF-8 particles. (a) Rhombic dodecahedral ZIF-8 particles. (b) Rhombic dodecahedral ZIF-8@SiO_2_ particles. (c) Cubic ZIF-8 and ZIF-8@SiO_2_ particles.

**Fig. S8.** XPS spectra of bare and SiO_2_-coated ZIF-8 particles. (a) Rhombic dodecahedral ZIF-8 particles. (b) Rhombic dodecahedral ZIF-8@SiO_2_ particles. (c) Cubic ZIF-8 and ZIF-8@SiO_2_ particles.

**Fig. S9.** Broadband acoustic emission analysis of representative samples. (a–c) Representative full PCD frequency spectra of water, 150 nm ZIF-8, and 150 nm ZIF-8@SiO_2_ at 1.67 MPa. (d) Relative broadband emission power as a function of acoustic pressure. Data in (d) are presented as mean ± SD from three independently synthesized batches (n = 3).

**Fig. S10.** Contact angle measurements of bare and SiO₂-coated ZIF-8 particles. (a–d) Rhombic dodecahedral ZIF-8 particles of 100, 150, 200, and 250 nm. (e–h) Rhombic dodecahedral ZIF-8@SiO_2_ particles of 100, 150, 200, and 250 nm. (i, j) Cubic ZIF-8 and ZIF-8@SiO_2_ particles.

**Fig. S11.** Post-ultrasound characterization of representative 150 nm rhombic ZIF-8@SiO_2_ nanoparticles. (a) TEM image and (b) DLS size distribution after ultrasound exposure.
